# Duel-stage treatment for biliary cysts with cholangitis during pregnancy

**DOI:** 10.12669/pjms.332.12148

**Published:** 2017

**Authors:** Baoxing Jia, Ludong Tan, Zhe Jin, Yahui Liu

**Affiliations:** 1 Baoxing Jia, Department of Hepatobiliary and Pancreatic Surgery, The First Hospital of Jilin University, Changchun, China; 2 Ludong Tan, Department of Hepatobiliary and Pancreatic Surgery, The First Hospital of Jilin University, Changchun, China; 3 Zhe Jin, Department of Hepatobiliary and Pancreatic Surgery, The First Hospital of Jilin University, Changchun, China; 4 Yahui Liu, Department of Hepatobiliary and Pancreatic Surgery, The First Hospital of Jilin University, Changchun, China

**Keywords:** Biliary cyst, Percutaneous cholecystostomy, Pregnancy

## Abstract

**Background & Objective:**

Biliary cysts in pregnant women are a complex medical issue, especially when complicated with cholangitis. It is a serious and life-threatening diagnosis that can seriously endanger both the expectant mother and the fetus. However, during pregnancy, surgical treatment would lead to further complications and higher fetal mortality. Here, we propose a novel therapeutic approach that would be safe for both mother and child during pregnancy, with a definitive treatment postponed until after delivery.

**Methods:**

In this retrospective study we have summarized the clinical course of six adult patients diagnosed with choledochal cysts during pregnancy. Treatment was conducted in two stages, firstly, percutaneous cholecystostomy under ultrasound guidance and sustained negative pressure suction until delivery, and secondly, selective choledochal cyst excision when the patients recovered from delivery.

**Results:**

All the six patients gave birth to healthy babies. Four patients had Type-I choledochal cysts, and underwent Roux-en-Y hepaticojejunostomy surgery. Two patients had a Type-IV choledochal cyst. The first patient with Type-IV choledochal cyst underwent anastomosis between the secondary hepatic bile duct and jejunum and the second patient underwent laparoscopic cyst internal drainage. No serious complications were recorded after gallbladder drainage or during the perioperative period.

**Conclusions:**

Based on our single-centre experience we can conclude that treatment of choledochal cyst with cholangitis during pregnancy can be conducted safely and efficiently through the two stages strategy that we proposed in this paper. The first stage should be percutaneous cholecystostomy followed by elective surgical treatment following delivery.

## INTRODUCTION

Biliary cysts are cystic malformations that may occur anywhere in the bile duct system. Based on the latest classification scheme, they include intra and extra hepatic biliary cysts as well as the presence of an abnormal pancreatobiliary junction. Previously, we distinguished five types of biliary cysts.^1^ Type I cysts are most common (50 to 85% of cases) and are characterized by cystic or fusiform dilation of the common bile duct. These can be further subcategorized in Type IA, type IB and type IC. Type II cysts are diverticula of the extrahepatic bile duct and represent 2% of cysts. Type- III cysts represent a group of cystic dilatations limited to the intraduodenal area of the distal common bile duct and are also subcategorized into type IIIA and type IIIB (1 to 5% of cysts). Type IV cysts (15 to 35% of cysts) are defined as multiple cysts that can have both intra and extrahepatic involvement (Type-IVA have both intra and extrahepatic involvement, type IVB have only extrahepatic cysts). Type-V cysts imply one or more intrahepatic dilatations. Prevalence rates show a marked female predominance, with a female to male ratio of 3:1.^2^ Most patients with biliary cysts will present in their childhood,^3^ by the age of 10 with only 20% of cases undiagnosed into adulthood. The clinical presentation most often consists of abdominal pain, palpable mass and jaundice.^4^ This clinical presentation is more typical for children than adults.^5^

Biliary cysts complicated with cholangitis in pregnant women are an extremely rare condition.^6^ To the best of our knowledge, there are no large or relevant epidemiological studies that show the prevalence of biliary cysts in pregnant women, or the prevalence of cholangitis within this group of patients. There are only several case-series or case reports available in recent literature. However, such cases are considered highly life-threatening for both expectant mothers and the fetus.^7^ Until now, there was no unified proposed treatment strategy for the treatment of choledochal cysts with cholangitis during pregnancy. In the past five years, we have developed an effective method, which provides a safe and efficacious approach to this complex clinical situation. Our goal was to ensure the safety of the mother and child until the end of pregnancy, followed by a complete removal of the lesion of the common bile duct post-delivery.

## METHODS

### Presentation of cases

From January 2010 to January 2015, we identified six in-patients with choledochal cysts alongside cholangitis during pregnancy. The age of the patients ranged from 20 to 27 with gestational age from 20 to 32 weeks. The patients presented to hospital due to varying degrees of upper abdominal pain, fever or jaundice. All of them underwent abdominal ultrasound demonstrating cystic dilatation of the common bile duct and inflammation. Blood samples were also taken for laboratory analysis. As patients were highly susceptible, they were immediately admitted for further treatment and evaluation.

### Treatment strategy

Upon admission, we conducted some routine medical tests including routine blood sampling, liver function test and electrolyte levels. Empirical antibiotic treatment was initiated as well as rapid fluid replacement. Considering the particularity of these cases, we consulted the help of the Department of Obstetrics and Gynecology at the time of admission. Based on the Tokyo guidelines for cholangitis,^8^ these six patients did not require emergency exploratory laparotomy. In order to ensure the safety of the expectant mother and fetus, we decided to treat them in stages. The first stage was percutaneous transhepatic gallbladder drainage (PTGBD) under ultrasound guidance and sustained negative pressure suction, which was maintained until delivery. The second staging was elective choledochal cyst excision after the patient recovered after delivery. The protocol and the consent form of this manuscript were approved by the institution’s Human Research Committee of the Jilin University, China.

## RESULTS

### First hospitalization

The clinical data of six patients collected during their first hospitalization are summarized in Table-I. After antibiotic treatment and rapid fluid infusion was completed, only one patient underwent MRCP in addition to the ultrasound check up due to the possible risks for the fetus. All patients had PTGBD conducted using a 22 G puncture tube of the COOK company, and biliary drainage for three to five days. Every day, approximately 400ml of bile was drained, and the symptoms of acute inflammation gradually relieved. After the symptoms improved significantly, we clipped the drainage tube and discharged these patients from the hospital. After delivery and recovery, patients came back and had the definitive surgery for the choledochal cyst. Only one patient returned, two weeks after discharge, due to frequent vomiting. We maintained the electrolyte balance combined with rapid fluid infusion. After the treatment, the symptoms of the patient relieved.

**Table-I:** First hospitalization information.

	*Patient I*	*Patient II*	*Patient III*	*Patient IV*	*Patient V*	*Patient VI*
Age (years)	20	23	24	27	27	27
Parity	Primipara	Primipara	Multipara	Primipara	Primipara	Primipara
Gestational weeks	28	30	20	32	22	26
Clinical symptoms	Upper abdominal pain, jaundice, fever	Upper abdominal pain, jaundice, fever	Upper abdominal pain, jaundice, vomiting	Upper abdominal pain, fever, nausea	Upper abdominal pain, fever, vomiting	upper abdominal pain, jaundice, fever
Leukocyte (×10^9^/L)	12.9	14.5	12.2	11.0	13.2	15.1
Total bilirubin (umol/L)	240	150	120	10.1	26.3	100
Ultrasound diagnosis	Not clear	Clear	Clear	Clear	Clear	Clear
Other imaging examination	MRCP	None	None	None	None	None
Imaging reveal	Cyst mass of common bile duct with internal density	Cystic dilatation of common and intrahepatic bile duct	Cystic dilatation of common bile duct	Cystic dilatation of common bile duct	Cystic dilatation of common bile duct	Cystic dilatation of common and intrahepatic bile duct
Cholelithiasis	Gallbladder and bile duct stones	Bile duct stones	None	None	None	Gallbladder and bile duct stones
Size of choledochal cyst (cm)	12×10	15×5	10×8	12×5	11×6	11×6
PTGBD	Successful	Successful	Successful	Successful	Successful	Successful

†***PTGBD*** percutaneous transhepatic gallbladder drainage

### Second hospitalization

The clinical data of the six patients collected during their second hospitalization are summarized in Table-II.

**Table-II:** Second hospitalization information.

	*Patient I*	*Patient II*	*Patient III*	*Patient IV*	*Patient V*	*Patient VI*
Admission date after delivery (days)	35	33	33	32	40	35
Delivery mode	Vaginal delivery	Vaginal delivery	Cesarean section	Cesarean section	Vaginal delivery	Vaginal delivery
Leukocyte (×10^9^/L)	7.7	6.6	5.9	8.8	6.5	9.0
Total bilirubin (umol/L)	24.5	15.4	17.9	13.5	12.2	12.8
Imaging examination	Ultrasound	Ultrasound, MRCP	Ultrasound, MRCP	Ultrasound, MRCP	Ultrasound, abdominal CT	Ultrasound, MRCP
Type of cyst	Type I	Type IV	Type I	Type I	Type I	Type IV
Operation method	Cyst resection, hepaticojejunostomy	Cyst resection, anastomosis between secondary hepatic bile duct and jejunum	Cyst resection, hepaticojejunostomy	Cyst resection, hepaticojejunostomy	Cyst resection, hepaticojejunostomy	laparoscopic cyst internal drainage
Postoperative complications	Intraperitoneal infection	None	None	None	Much intraperitoneal drainage	None

Patients came back to our department after they delivered healthy babies one month later. All of the patients had no infection, no electrolyte disorder and bilirubin levels within normal range. However, all patients had mildly elevated liver enzymes. Patients were followed up with imaging tests, defining the classification of their biliary cysts and made pre-operative plans.

### The prognosis and follow-up

There were no serious complications after drainage and during the perioperative period. We followed up all six patients six months to one year, and no serious complications were recorded. The immediate curative effect was excellent.

## DISCUSSION

The diagnostic rate of adult choledochal cyst accounts for only around 20% of cases in the literature. Even though large studies or systematic reviews regarding such patients are scarce, it is safe to conclude that choledochal cysts are rarely diagnosed during pregnancy. Biliary cysts represent a life-threatening diagnosis for both expectant mother and the fetus, when complicated with cholangitis. During pregnancy, the intra-abdominal pressure increases.^9^ This may disrupt the dynamic balance of the static choledochal cyst, resulting in some severe complications. Due to the pathophysiological processes involved, one of the most common underlying diseases is cholangitis. For a clinician, it is difficult to guarantee successful outcomes if the resection of lesions and bile duct is conducted in the first admission. Therefore, choosing a reasonable operation time and operative methods is the key to the success of the procedure.

In 1977, Todani^10^ classified choledochal cyst into five types. Most surgeons apply this classification clinically. The operative methodology has been explored in the last few decades. These have included open internal or external cyst drainage, Roux-en-Y hepaticojejunostomy, and liver transplantation for Caroli’s disease.^11^

Although the traditional operative technique is suitable for the mother, it inevitably has many adverse impacts on delivery and the fetus. According to some studies, surgery during pregnancy, increases significantly mortality and complication rates.

Other studies have demonstrated that laparotomy and internal or external drainage in the second trimester of pregnancy was relatively safe.^12^ Nevertheless, there have been reported cases resulting in premature delivery under surgical intervention. Based on the specificity of choledochal cysts with cholangitis during pregnancy, it is important to develop and explore a method which can ensure minimal trauma to both the expectant mother and fetus. Guided by the concept of minimally invasive treatment, we sought to devise a procedure that would control and cure the disease whilst reducing mortality and complications rates. In this study we have demonstrated a treatment by stages, instead of a traditional one-stage operation, aimed at improving operative outcomes.

After diagnosis has been made within 24 hours, we conducted PTGBD under ultrasound guidance and sustained negative pressure suction in our patients. Due to inflammation of the common bile duct, the pressure in the bile duct was higher than in the gallbladder. The bile wall was sucked by external negative pressure drainage device according to the pressure distribution. The reason why we opted for PTGBD instead of percutaneous transhepatic bile duct drainage (PTBD) or other choledochal cyst drainage is that PTGBD is more convenient for emergency treatment. Additionally, its functionality can also be easily restored if the tube becomes blocked or loose during drainage. Furthermore, the fetus is minimally endangered during PTGBD.

The symptoms of cholangitis can be relieved within 24 - 48 hours after drainage. We observed these patients for three to five days after the procedure, while maintaining the balance of electrolytes combined with rapid fluid infusion. When the patients were in stable condition, patients were discharged after the drainage tube was clipped.

Nearly one month after delivery, six patients came back to our department and completed examinations. Four of them had Type-I choledochal cysts, and underwent Roux-en-Y hepaticojejunostomy. The operations were successful and no unexpected events were reported. One patient was diagnosed with Type-IV choledochal cyst. We decided for an anastomosis between the secondary hepatic bile duct and jejunum using the Roux-en-Y method. Because of partial cyst excision, some intrahepatic biliary cysts remained. Due to personal reasons, another patient with Type-IV choledochal cyst refused an operation, so we conducted a laparoscopic cyst internal drainage.

After surgery, one patient with a larger cyst and a long operation time had a persistent low fever and right upper abdominal pain. We assumed that he may have an intraperitoneal infection, and after antimicrobial treatment the symptoms relieved. Another patient’s cyst adhered to surrounding tissue tightly which resulted in intraoperative bleeding. The total volume of intraperitoneal drainage was increased. After conservative treatment, their condition improved. Those postoperative complications match to Grade II of Clavien-Dindo classification.^13^

In recent years, minimally invasive treatment has become the forefront of treating conditions with minimal complications. Due to the possible severe complications^14^-^16^ for expectant mothers and fetuses, it was unreasonable to conduct surgical resection and exploration immediately after the diagnosis has been made. Without a thoroughly conducted examination prior to the surgery it is difficult to create a detailed operation plan.

This study showed that PTGBD, under ultrasound guidance, could relieve the symptoms of cholangitis and have minimal impact on the delivery and fetus. At the same time, it reduced pressure in the bile duct and avoided severe complications such as ruptured cholangitis. After the relief of cholangitis, patients were able to return home for recovery with a clipped drainage tube. If the symptoms of cholangitis emerged again during the period of pregnancy, the patient could re-open the drainage tube. This convenience had a significant advantage over open or laparoscopic cyst drainage. Another benefit of this strategy was that doctors were able to diagnose the cyst type followed by an optimal surgical plan. It is possible that long-term biliary drainage interfered with bile metabolism, as the liver enzymes of the patients slightly increased. After the operation, two patients developed mild complications which were cured soon. We consider those complication completely unrelated to our treatment by stages.

## CONCLUSIONS

We can conclude that choledochal cysts with cholangitis during pregnancy are a complicated disease to treat. Our treatment by stages approach, presented in this study, represents a treatment with optimal efficacy and without severe complications.

### Limitations of the Study

There are some limitations of this study. It represents a case series of six patients which makes these results unsuitable for a definite conclusion. Larger studies that would support our approach are needed. Moreover, we should conduct more studies regarding the liver function, electrolyte imbalance and other pathophysiological processes present in these patients.

### Authors’ Contribution

**Baoxing Jia:** Conception and design of study.

**Ludong Tan:** Acquisition of data and drafting of manuscript.

**Zhe Jin:** Data analysis.

**Yahui Liu:** Conception and design of study.
